# Computer vision and augmented reality in gastrointestinal endoscopy

**DOI:** 10.1093/gastro/gov027

**Published:** 2015-06-29

**Authors:** Nadim Mahmud, Jonah Cohen, Kleovoulos Tsourides, Tyler M. Berzin

**Affiliations:** ^1^Department of Internal Medicine, Brigham and Women’s Hospital, Harvard Medical School, Boston MA, USA; ^2^The Center for Advanced Endoscopy, Beth Israel Deaconess Medical Center, Harvard Medical School, Boston MA, USA; ^3^Brain and Cognitive Sciences, Massachusetts Institute of Technology, Boston MA, USA

**Keywords:** augmented reality, gastrointestinal endoscopy, colonoscopy, computer vision

## Abstract

Augmented reality (AR) is an environment-enhancing technology, widely applied in the computer sciences, which has only recently begun to permeate the medical field. Gastrointestinal endoscopy—which relies on the integration of high-definition video data with pathologic correlates—requires endoscopists to assimilate and process a tremendous amount of data in real time. We believe that AR is well positioned to provide computer-guided assistance with a wide variety of endoscopic applications, beginning with polyp detection. In this article, we review the principles of AR, describe its potential integration into an endoscopy set-up, and envisage a series of novel uses. With close collaboration between physicians and computer scientists, AR promises to contribute significant improvements to the field of endoscopy.

## Introduction

Augmented reality (AR) is the process of superimposing computer-generated objects and data over existing, real structures [1]. This differs from virtual reality, where the basic elements of the environment are entirely computer-generated in an effort to simulate their existence [2]. Augmentation typically operates within the semantic context of environmental elements. A simple example of this is displaying ‘live’ scores on a televised sports match. With the help of advanced AR technology, such as computer vision and object recognition, information about the environment can become interactive and easier to manipulate from a computational perspective [3–6]. AR has empirically sought to seamlessly integrate reality with analytical information, to improve a user’s ability to perform a task in real time. Consider, for example, the earliest application of AR when Mark VIII fighters in World War II had live radar information displayed in the pilot's line of sight. This improved the pilot’s ability to locate other airplanes in the sky and identify enemy aircraft [7].

Despite its long history, AR has only recently made its debut in medical practice, being applied primarily to navigational surgery [8]. This involves taking data from pre-operative imaging and using anatomical anchors in the operating field to link—or register—the two representations in real time. The neurosurgical and otolaryngological fields have used AR to map 3D images of a patient’s paranasal sinus or neuroanatomy on monitors, and mobile devices to assist with various surgical procedures, including prototypes to display the ventricular system for drain placement and brain tumors for resection planning [9, 10]. Cabrilo *et al.* utilized AR in 33 patients, using representations of the cerebral arteries to assist with arteriovenous malformation resection and aneurysm clipping [11, 12]. Finally, several studies describe landmark-based AR systems for endoscopic sinus or skull-base surgeries [13–15].

Although few studies in general surgery have taken AR to the operating room, environments have been developed for both laparoscopic and open procedures [16]. López-Mir *et al.* used AR to improve the accuracy of trocar placement for cholecystecomy [17], while Kang *et al.* integrated laparoscopic ultrasound imaging in order to interrogate the liver, gallbladder, biliary tree, and kidneys below the visible surface [18]. Volonte *et al.* reported a successful cholecystectomy using AR-based stereoscopic images with the da Vinci robot [19], and Simpfendörfer *et al.* generated AR images from transrectal ultrasound to perform a radical prostatectomy [20]. The demand for AR in open surgeries has been more limited, but Peterhans *et al.* performed partial hepatic resections in nine patients, with AR used to display vascular and biliary anatomy [21], and Marzano *et al.* completed a pancreaticoduodenectomy using AR to highlight important vascular structures [22].

The role of AR in gastrointestinal (GI) endoscopy to date has been limited to localization of GI tumors and novel transluminal surgical approaches. Kranzfelder *et al.* described an AR system that successfully registered CT data with upper endoscopy imaging in 24 patients with GI tumors [23]. Azagury *et al.* developed an image registration (IR) system to assist with natural orifice transluminal endoscopic surgery (NOTES) [24]. IR-NOTES was used to target various intra-abdominal organs with the endoscope in 15 cadavers. This group and Vosburgh *et al.* found that easy transluminal access to the kidneys, gallbladder, liver, and pancreas could be achieved with close AR guidance, raising the possibility of novel endoscopic surgical procedures [24, 25].

While the potential of navigational surgery is exciting, we believe that GI endoscopy is fertile ground for a vast array of new applications, including detection and identification of polyps, performance tracking, pathology scoring and more. In this article we review the components of AR systems, elaborate on our proposed applications for AR, highlight technical challenges, and outline a path towards innovation.

## Technical considerations for AR in the endoscopy unit

Important to the fundamentals of AR are the technologies of image processing and computer vision. Image processing refers to the deconstruction of image data into a series of parameters or properties relating to the image. ‘Computer vision’ refers to high-level image processing in which a computer deciphers the contents of an image or sequence of images and uses this information to make intelligent decisions [26–29]. In essence, these technologies allow computers to ‘see and understand’ environments, and to make complex judgments for further output [30]. In the case of video- and image enhancement through graphical overlay, the output is termed ‘augmented reality’.

There is a wide variety of AR set-ups and applications but they have in common a data input source, a processor, and a display [31]. The input source is commonly a camera, which provides information for the computer to compare with image databanks, effectively allowing it to ‘see’ what the user sees. The display is the medium for combining reality with virtual information. In different clinical settings, the display may be a standard video monitor or an optical head-mounted display, Google Glass being a recent example of the latter.

The basic hardware set-up for an endoscopy procedure is well suited to adaptation to an AR environment because high-definition video capture is already a core capability of most modern endoscopy units. A high-definition camera, at the distal tip of the flexible endoscope, supplies image data to a camera control unit (CCU), where the data are processed and formatted for output to high-definition monitors. The CCU is further connected to a quick-swap memory unit and a computer or keyboard to accept user input. An added central processing unit can be inserted in series between the CCU and monitor, to enable image processing and computer vision capabilities. This will house the signal processing, image analysis, and decision-making capabilities of the system prior to modified high-definition output with graphical overlay.

## AR to improve polyp detection

Colonoscopy is the ‘gold standard’ for early detection of colorectal adenomas [32], and more than 14 million colonoscopies are performed in the United States annually [33]. Despite this volume, there is a significant adenoma miss rate (AMR), according to the literature ranging from 6–27% and depending on a variety of polyp and operator characteristics [34]; for example, smaller polyps [26, 35], flat polyps [37], and left colonic location [38] may be associated with an increased miss rate. Sessile serrated adenomas are a topic of particular concern, given their higher predilection towards neoplastic change [39, 40] and, unlike pedunculated polyps, are more frequently missed in the right side of the colon [41]. Operator experience and fatigue are also significant considerations; endoscopists are more likely to miss polyps in the afternoon, as compared to morning cases [42]. Importantly, there is convincing evidence that having ‘more eyes’ on the video monitor increases the adenoma detection rate. Observation of the video monitor by nurses has been shown to increase polyp detection by ∼30% [43, 44], and participation by a gastroenterology trainee has been shown to do the same [45]. These findings suggest that individual endoscopists routinely miss polyps that are visible in the monitor.

Increasing adenoma detection rate improves the preventive efficacy of colonoscopy and polypectomy [46, 47]. During a colonoscopy, there are two general reasons why a polyp might be missed: (i) it was never in the visual field or (ii) it was in the visual field but not recognized.

Several hardware innovations have sought to address the first problem through expanded visualization of the colonic lumen. This includes the Third Eye retroscope camera, designed to identify polyps hidden behind folds in the bowel wall [48], and full-spectrum endoscopy (FUSE), which provides a wider, 330° left-to-right endoscopic view [49]. The second problem—unrecognized polyps within the visual field—has been more difficult to address. In addition to the available data on the benefit, in terms of polyp detection, of additional observers in the endoscopy suite, multiple studies have attempted to use chromoendoscopic dyes [50–52] or narrow-band imaging [53] to make flat or isochromatic polyps more apparent. There exists a great deal of controversy surrounding the comparative effectiveness of these approaches [54].

**Figure 1.**
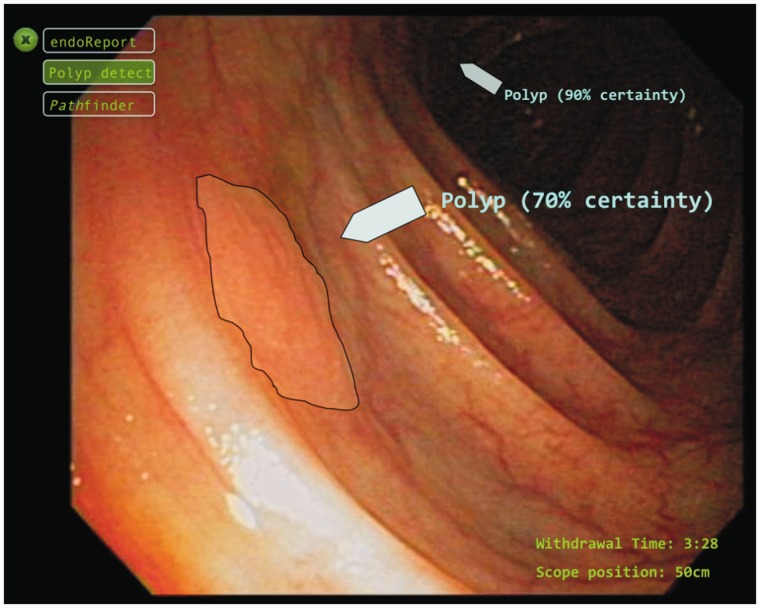
An illustrative example of augmented reality-assisted polyp recognition with graphic overlay on the endoscopy screen. The flat lesion outlined represents a presumed flat polyp as detected by the computer vision algorithm. [Source: EndoLayers]

Augmented reality represents an important opportunity to improve adenoma detection rate. By aggregating a large volume of polyp images from colonoscopies, it is possible to implement machine-learning and computer vision algorithms to assist with polyp detection. This would be the first line of approach to innovation within the context of the high-resolution video data that is routinely acquired during colonoscopy. Using AR, the endoscopist could enjoy real-time visual assistance with polyp detection in the form of overlaid images on the primary HD monitor or on an adjacent one. In more advanced iterations, suspected polyp type may also be displayed, with color-coding or other visual information used to represent the level of confidence in the analysis (Figure 1).

Several important features of this technology can be highlighted. First, modern processing capabilities would enable an AR system to function in real time during a colonoscopy. Second, flat or isochromatic polyps, which might be visually occult to the less experienced or fatigued endoscopist, would be parsed by the AR system in identical fashion to any other polyp; that is to say that, while humans have a tendency to “see what they want to see” [55], an AR system will “see what it is trained to see” with fidelity and without bias. Third, as more image data is acquired for analysis, the AR system will become increasingly efficient and accurate in detecting polyps of all varieties.

Finally, AR is complementary to contemporary hardware innovations designed to improve adenoma detection rate through expanded visualization. Indeed, the Third Eye and FUSE systems involve additional cameras and force the operator to scan multiple screens displaying live video. As mentioned above, AR could draw the endoscopist’s eye, in real time, to lesions that give rise to concern, effectively creating ‘extra sets of eyes’ on all aspects of the video data.

## Other potential AR applications in GI endoscopy

### Computer vision for polyp classification

The American Society for Gastrointestinal Endoscopy recommends that all neoplastic polyps in the colon be resected [56]; however, distinguishing between neoplastic and non-neoplastic polyps can be extremely challenging at the time of endoscopy. Up to 35% of colonic polyps are non-neoplastic, including hyperplastic, inflammatory, and mucosal polyps [57]. Polypectomy followed by histopathological analysis of all acquired specimens is currently the ‘gold standard’ approach to polyp classification. To minimize unnecessary polyp resection—which prolongs procedures and increases the risk of morbidity—several novel technologies have emerged to distinguish neoplastic from non-neoplastic polyps. These include narrow-band imaging and endocytoscopy, both of which require specialized training and additional hardware [58–60]. Definitive polyp classification through AR could provide similar benefit with less expense. As detailed above, AR can work with existing endoscopy set-ups and would require minimal training.

### Multimodality image enhancement

There are several imaging techniques used during gastrointestinal endoscopy, which could benefit from integration with augmented reality. Endoscopic ultrasound, for example, can be used to characterize organs or masses adjacent to the gastrointestinal tract. Structural images obtained using EUS could be registered in order to display a spatial projection of the object in the live colonoscopy video feed. Registration of EUS images for computer-guided navigation has previously been described [61], and could simplify targeting for needle biopsies or cyst drainage. Similarly, in endoscopic retrograde cholangiopancreatography (ERCP), both fluoroscopic and endoscopic images are used simultaneously during evaluation of the pancreaticobiliary system. Applying AR solutions to fluoroscopic images has been explored in the arena of cardiac interventions [62], and image analysis tools to measure the diameter and length of fluoroscopically identified biliary strictures might facilitate accurate stent selection for optimal treatment of strictures.

### Performance improvement and tracking

As governmental and insurance regulations surrounding accountable care take form, there will be ever-increasing pressure for endoscopists to track their polyp detection rates and other performance metrics; thus another critical aspect of endoscopic AR could be to track, test, and validate polyp detection performance. This could assist in quality reporting for the purpose of tracking and improving outcomes in gastrointestinal endoscopy [63]. Tracking could also generate automated data regarding colonoscopy withdrawal time, which has been significantly linked to the quality of adenoma detection [64]. This information could be archived in order to inform endoscopists about performance measures in real time, as well as during aggregate performance reviews.

### Digital ruler for improved accuracy of measurement

Accurate estimation of the size of various anatomical structures during endoscopy has the potential to greatly enhance current practice. In the arena of polyp detection, determining the size of a given polyp plays a critical role in dictating follow-up intervals for a patient’s next surveillance colonoscopy. Recent data suggest that, with visual estimation, substantial variations in recorded polyp size occur, leading to incorrect surveillance intervals in 10% of cases [65, 66]. To date there is no digital tool to assist with polyp sizing and traditional methods, such as comparison with biopsy forceps and snares, add little to accuracy [67]. While a ‘digital ruler’ using AR might require a second camera, several endoscopes already incorporate multiple lenses, and innovative reference points may ultimately enable accurate measurement with standard single-camera endoscopes.

### Standardized scoring/grading systems

Standardized endoscopic scoring systems exist for many disease states of the gastrointestinal tract. Unfortunately, their utility is often limited by subjective interpretation and impracticality in the clinical setting. Inflammatory bowel disease (IBD) scoring systems, for example, are used to assess disease response in clinical trials but have been difficult to apply in clinical practice [68, 69]. AR-assisted IBD scoring could determine patient response to novel therapeutics through objective assessment of mucosal healing. A second application that would benefit from objective scoring is the grading of bowel preparation quality. Despite its critical importance in informing surveillance intervals, current grading is operator-dependent and highly variable [70, 71]. AR-based scales, based on stool burden, would minimize subjectivity and globally improve adherence to surveillance guidelines.

### Dynamic braking for capsule endoscopy

Wireless capsule endoscopy is indicated to interrogate portions of the small bowel that are not easily accessed through standard endoscopy—usually to search for obscure gastrointestinal bleeding or signs of Crohn’s disease. Recent advances have granted some degree of control over maneuvering the capsule whilst it is in the gastrointestinal tract. This has been accomplished through techniques such as electro-stimulation and by using magnetic fields [72, 73]. Although video data from wireless capsules are currently processed after completion of the endoscopy, it is likely that future generations of capsules will support real-time video analysis. When this is available, computer vision and augmented reality could be used to automatically detect pathology and trigger slowing of the capsule using the methods noted above. This would maximize the video data acquired to better delineate lesions of interest.

## The road ahead

While no software platforms to integrate augmented reality into endoscopy are currently available, this is an active area for research and innovation. We have only just entered into an era in which computer vision technology may surpass human ability in terms of facial and object recognition [74]. The combination of computer vision and endoscopy thus offers vast potential to enhance the abilities of the average endoscopist. For this technology to integrate into regular practice, however, numerous challenges must be overcome. First, in order to optimize the signal–noise ratio and minimize false positive (during polyp recognition, for example), enormous image and video repositories will be needed for reference and machine learning. Second, careful quality control must be paramount in all aspects of novel AR applications. Finally, creating a useful AR assistance tool for endoscopists will necessarily require close collaboration between clinicians, software engineers and computer vision experts. Careful attention to these potential pitfalls may bring AR to the forefront of the next era of gastrointestinal endoscopy.

*Conflict of interest statement*: NM, JC, KT, and TB are advisors to EndoLayers.

## References

[1] Tang SL Kwoh CK Teo MY Augmented reality systems for medical applications. IEEE Eng Med Biol Mag 1998;17:49–58. 9604701 10.1109/51.677169

[2] Wright WG Using virtual reality to augment perception, enhance sensorimotor adaptation, and change our minds. Front Syst Neurosci 2014;8:56. 24782724 10.3389/fnsys.2014.00056 PMC3986528

[3] Graham M Zook M Boulton A Augmented reality in urban places: contested content and the duplicity of code. Trans Inst Br Geogr 2013;38:464–79.

[4] Chen BX If you’re not seeing data, you’re not seeing. Wired Magazine 2009 August 25.

[5] Maxwell K Augmented Reality. Macmillan Dictionary Buzzword, 2010.

[6] Azuma RT A survey of augmented reality. Presence 1997;6:355–85.

[7] Vaughan-Nichols SJ Augmented reality: No longer a novelty? Computer 2009;42:19–22.

[8] Shuhaiber JH Augmented reality in surgery. Arch Surg 2004;139:170–4. 14769575 10.1001/archsurg.139.2.170

[9] Kramers M Armstrong R Bakhshmand SM Evaluation of a mobile augmented reality application for image guidance of neurosurgical interventions. Stud Health Technol Inform 2014;196:204–8. 24732507

[10] Mahvash M Tabrizi LB A novel augmented reality system of image projection for image-guided neurosurgery. Acta Neurochir (Wien) 2013;155:943–7. 23494133 10.1007/s00701-013-1668-2

[11] Cabrilo I Bijlenga P Schaller K Augmented reality in the surgery of cerebral arteriovenous malformations: technique assessment and considerations. Acta Neurochir (Wien) 2014;156:1769–74. 25037466 10.1007/s00701-014-2183-9

[12] Cabrilo I Bijlenga P Schaller K Augmented reality in the surgery of cerebral aneurysms: a technical report. Neurosurgery 2014;10 Suppl 2:252–61. 24594927 10.1227/NEU.0000000000000328

[13] Thoranaghatte RU Giraldez JG Zheng G Landmark based augmented reality endoscope system for sinus and skull-base surgeries, In Engineering in Medicine and Biology Society, 2008. EMBS 2008. 30th Annual International Conference of the IEEE, IEEE, 2008. 10.1109/IEMBS.2008.4649094 19162597

[14] Thoranaghatte R Garcia J Caversaccio M Landmark‐based augmented reality system for paranasal and transnasal endoscopic surgeries. Int J Med Robot 2009;5:415–22. 19623600 10.1002/rcs.273

[15] Caversaccio M Langlotz F Nolte LP Impact of a self-developed planning and self-constructed navigation system on skull base surgery: 10 years experience. Acta Otolaryngol 2007;127:403–7. 17453461 10.1080/00016480601002104

[16] Okamoto T Onda S Yanaga K Clinical application of navigation surgery using augmented reality in the abdominal field. Surg Today 2015;45:397–406. 24898629 10.1007/s00595-014-0946-9

[17] López-Mir F Naranjo V Fuertes J Design and validation of an augmented reality system for laparoscopic surgery in a real environment. Biomed Res Int 2013;2013:758491. 24236293 10.1155/2013/758491 PMC3819885

[18] Kang X Azizian M Wilson E Stereoscopic augmented reality for laparoscopic surgery. Surg Endosc 2014;28:2227–35. 24488352 10.1007/s00464-014-3433-x

[19] Volonté F Buchs NC Pugin F Stereoscopic augmented reality for da Vincii^™^ robotic biliary surgery. Int J Surg Case Rep 2013;4:365–7. 23466685 10.1016/j.ijscr.2013.01.021 PMC3605472

[20] Simpfendörfer T Baumhauer M Müller M Augmented reality visualization during laparoscopic radical prostatectomy. J Endourol 2011;25:1841–5. 21970336 10.1089/end.2010.0724

[21] Peterhans M vom Berg A Dagon B A navigation system for open liver surgery: design, workflow and first clinical applications. Int J Med Robot 2011;7:7–16. 21341357 10.1002/rcs.360

[22] Marzano E Piardi T Soler L Augmented reality-guided artery-first pancreatico-duodenectomy. J Gastrointest Surg 2013;17:1980–3. 23943389 10.1007/s11605-013-2307-1

[23] Kranzfelder M Wilhelm D Doundoulakis M A probe-based electromagnetic navigation system to integrate computed tomography during upper gastrointestinal endoscopy. Endoscopy 2014;46:302–5. 24254384 10.1055/s-0033-1358814

[24] Azagury D Ryou M Shaikh S Real‐time computed tomography‐based augmented reality for natural orifice transluminal endoscopic surgery navigation. Br J Surg 2012;99:1246–53. 22864885 10.1002/bjs.8838 PMC3677565

[25] Vosburgh KG San José Estépar R Natural Orifice Transluminal Endoscopic Surgery (NOTES): an opportunity for augmented reality guidance. Stud Health Technol Inform 2006;125:485–90. 17377333

[26] Marr D Vision: A computational investigation into the human representation and processing of visual information. New York, NY: Henry Holt and Co. Inc, 1982:2–46.

[27] Rosenfeld A Avinash C KAK: Digital Picture Processing. Academic Press, 1982.

[28] Barghout L Lee L Perceptual information processing system. Google Patents, 2003.

[29] Horn B Robot vision. MIT press, 1986.

[30] Faugeras O Three-dimensional computer vision: a geometric viewpoint. MIT press, 1993.

[31] Berryman DR Augmented reality: a review. Med Ref Serv Q 2012;31:212–18. 22559183 10.1080/02763869.2012.670604

[32] Schrock TR Colonoscopy versus barium enema in the diagnosis of colorectal cancers and polyps. Philadelphia, PA: WB Saunders Co, 1995:513–38.

[33] Seeff LC Richards TB Shapiro JA How many endoscopies are performed for colorectal cancer screening? Results from CDC’s survey of endoscopic capacity. Gastroenterology 2004;127:1670–7. 15578503 10.1053/j.gastro.2004.09.051

[34] Ahn SB Han DS Bae JH The miss rate for colorectal adenoma determined by quality-adjusted, back-to-back colonoscopies. Gut Liver 2012;6:64–70. 22375173 10.5009/gnl.2012.6.1.64 PMC3286741

[35] Rex DK Cutler CS Lemmel GT Colonoscopic miss rates of adenomas determined by back-to-back colonoscopies. Gastroenterology 1997;112:24–8. 8978338 10.1016/s0016-5085(97)70214-2

[36] Postic G Lewin D Bickerstaff C Colonoscopic miss rates determined by direct comparison of colonoscopy with colon resection specimens. Am J Gastroenterol 2002;97:3182–5. 12492208 10.1111/j.1572-0241.2002.07128.x

[37] Heresbach D Barrioz T Lapalus M Miss rate for colorectal neoplastic polyps: a prospective multicenter study of back-to-back video colonoscopies. Endoscopy 2008;40:284–90. 18389446 10.1055/s-2007-995618

[38] Leufkens A van Oijen M Vleggaar F Factors influencing the miss rate of polyps in a back-to-back colonoscopy study. Endoscopy 2012;44:470–5. 22441756 10.1055/s-0031-1291666

[39] Hurlstone DP Cross SS Adam I A prospective clinicopathological and endoscopic evaluation of flat and depressed colorectal lesions in the United Kingdom. Am J Gastroenterol 2003;98:2543–9. 14638361 10.1111/j.1572-0241.2003.07679.x

[40] Parra-Blanco A Gimeno-García AZ Nicolás-Pérez D Risk for high-grade dysplasia or invasive carcinoma in colorectal flat adenomas in a Spanish population. Gastroenterol Hepatol 2006;29:602–9. 17198636 10.1016/s0210-5705(06)71700-9

[41] Xiang L Zhan Q Zhao XH Risk factors associated with missed colorectal flat adenoma: a multicenter retrospective tandem colonoscopy study. World J Gastroenterol 2014;20:10927–37. 25152596 10.3748/wjg.v20.i31.10927 PMC4138473

[42] Choi HN Kim HH Oh JS Factors influencing the miss rate of polyps in a tandem colonoscopy study. Korean J Gastroenterol 2014;64:24–30. 25073668 10.4166/kjg.2014.64.1.24

[43] Aslanian HR Shieh FK Chan FW Nurse observation during colonoscopy increases polyp detection: a randomized prospective study. Am J Gastroenterol 2013;108:166–72. 23381064 10.1038/ajg.2012.237

[44] Lee CK Park DI Lee SH Participation by experienced endoscopy nurses increases the detection rate of colon polyps during a screening colonoscopy: a multicenter, prospective, randomized study. Gastrointest Endosc 2011;74:1094–102. 21889137 10.1016/j.gie.2011.06.033

[45] Buchner AM Shahid MW Heckman MG Trainee participation is associated with increased small adenoma detection. Gastrointest Endosc 2011;73:1223–31. 21481861 10.1016/j.gie.2011.01.060

[46] Zauber AG Winawer SJ O'Brien MJ Colonoscopic polypectomy and long-term prevention of colorectal-cancer deaths. N Engl J Med 2012;366:687–96. 22356322 10.1056/NEJMoa1100370 PMC3322371

[47] Jacob BJ Moineddin R Sutradhar R Effect of colonoscopy on colorectal cancer incidence and mortality: an instrumental variable analysis. Gastrointest Endosc 2012;76:355–64.e1. 22658386 10.1016/j.gie.2012.03.247

[48] Siersema PD Rastogi A Leufkens AM Retrograde-viewing device improves adenoma detection rate in colonoscopies for surveillance and diagnostic workup. World J Gastroenterol 2012;18:3400–8. 22807609 10.3748/wjg.v18.i26.3400 PMC3396192

[49] Gralnek IM Segol O Suissa A A prospective cohort study evaluating a novel colonoscopy platform featuring full-spectrum endoscopy. Endoscopy 2013;45:697–702. 23939509 10.1055/s-0033-1344395

[50] Kiesslich R Fritsch J Holtmann M Methylene blue-aided chromoendoscopy for the detection of intraepithelial neoplasia and colon cancer in ulcerative colitis. Gastroenterology 2003;124:880–8. 12671882 10.1053/gast.2003.50146

[51] Hurlstone DP Karajeh M Cross SS The role of high-magnification-chromoscopic colonoscopy in hereditary nonpolyposis colorectal cancer screening: a prospective “back-to-back” endoscopic study. Am J Gastroenterol 2005;100:2167–73. 16181364 10.1111/j.1572-0241.2005.41481.x

[52] Longcroft-Wheaton G Brown J Cowlishaw D High-definition vs. standard-definition endoscopy with indigo carmine for the in vivo diagnosis of colonic polyps. United European Gastroenterol J 2013;1:425–9. 10.1177/2050640613502963 PMC4040745 24917993

[53] Uraoka T Saito Y Matsuda T Detectability of colorectal neoplastic lesions using a narrow‐band imaging system: A pilot study. J Gastroenterol Hepatol 2008;23:1810–15. 19032454 10.1111/j.1440-1746.2008.05635.x

[54] Wallace MB Improving colorectal adenoma detection: technology or technique? Gastroenterology 2007;132:1221–3. 17408636 10.1053/j.gastro.2007.03.017

[55] Yotsumoto Y Sekuler R Out of mind, but not out of sight: Intentional control of visual memory. Mem Cogn 2006;34:776–86. 10.3758/bf03193425 17063909

[56] Davila RE Rajan E Baron TH ASGE guideline: colorectal cancer screening and surveillance. Gastrointest Endosc 2006;63:546–57. 16564851 10.1016/j.gie.2006.02.002

[57] Huang CS O'Brien MJ Yang S Hyperplastic polyps, serrated adenomas, and the serrated polyp neoplasia pathway. Am J Gastroenterol 2004;99:2242–55. 15555008 10.1111/j.1572-0241.2004.40131.x

[58] Rastogi A Optical diagnosis of small colorectal polyp histology with high-definition colonoscopy using narrow band imaging. Clin Endosc 2013;46:120–9. 23614121 10.5946/ce.2013.46.2.120 PMC3630305

[59] Takemura Y Yoshida S Tanaka S Computer-aided system for predicting the histology of colorectal tumors by using narrow-band imaging magnifying colonoscopy (with video). Gastrointest Endosc 2012;75:179–85. 22196816 10.1016/j.gie.2011.08.051

[60] Mori Y Kudo SE Wakamura K Novel computer-aided diagnostic system for colorectal lesions by using endocytoscopy (with videos). Gastrointest Endosc 2015;81:621–9. 25440671 10.1016/j.gie.2014.09.008

[61] Hummel J Figl M Bax M 2D/3D registration of endoscopic ultrasound to CT volume data. Phys Med Biol 2008;53:4303–16. 18653922 10.1088/0031-9155/53/16/006

[62] Chen Y Kwok KW Ge J Augmented reality for improving catheterization in magnetic resonance imaging-guided cardiac electrophysiology therapy. J Med Dev 2014;8:020917.

[63] Kappelman MD Dorn SD Peterson E Quality of care for gastrointestinal conditions: a primer for gastroenterologists. Am J Gastroenterol 2011;106:1182–7. 21731014 10.1038/ajg.2011.118

[64] Rex DK Optimal withdrawal and examination in colonoscopy. Gastroenterol Clin North Am 2013;42:429–42. 23931852 10.1016/j.gtc.2013.05.009

[65] Chaptini L Chaaya A Depalma F Variation in polyp size estimation among endoscopists and impact on surveillance intervals. Gastrointest Endosc 2014;80:652–9. 24679658 10.1016/j.gie.2014.01.053

[66] Eichenseer PJ Dhanekula R Jakate S Endoscopic mis-sizing of polyps changes colorectal cancer surveillance recommendations. Dis Colon Rectum 2013;56:315–21. 23392145 10.1097/DCR.0b013e31826dd138

[67] Kume K Watanabe T Yoshikawa I Endoscopic measurement of polyp size using a novel calibrated hood. Gastroenterol Res Pract 2014;2014:714294. 25093021 10.1155/2014/714294 PMC4100290

[68] Ferrante M Colombel JF Sandborn WJ Validation of endoscopic activity scores in patients with Crohn's disease based on a post hoc analysis of data from SONIC. Gastroenterology 2013;145:978–86.e5. 23954314 10.1053/j.gastro.2013.08.010

[69] Ahmad H Berzin TM Yu HJ Central endoscopy reads in inflammatory bowel disease clinical trials: The role of the imaging core lab. Gastroenterol Rep (Oxf) 2014;2:201–6. 24994835 10.1093/gastro/gou033 PMC4124272

[70] Calderwood AH Schroy PC 3rd Lieberman DA Boston Bowel Preparation Scale scores provide a standardized definition of adequate for describing bowel cleanliness. Gastrointest Endosc 2014;80:269–76. 24629422 10.1016/j.gie.2014.01.031 PMC4104141

[71] Kim EJ Park YI Kim YS A Korean experience of the use of Boston bowel preparation scale: a valid and reliable instrument for colonoscopy-oriented research. Saudi J Gastroenterol 2014;20:219–24. 25038207 10.4103/1319-3767.136950 PMC4131304

[72] Keller J Fibbe C Rosien U Recent advances in capsule endoscopy: development of maneuverable capsules. Expert Rev Gastroenterol Hepatol 2012;6:561–6. 23061707 10.1586/egh.12.26

[73] Swain P The future of wireless capsule endoscopy. World J Gastroenterol 2008;14:4142–5. 18636658 10.3748/wjg.14.4142 PMC2725374

[74] Taigman Y Yang M Ranzato MA Deepface: Closing the gap to human-level performance in face verification, In Computer Vision and Pattern Recognition (CVPR), 2014 IEEE Conference on, IEEE, 2014.

